# Science‐based Targets for Antibiotics in Receiving Waters from Pharmaceutical Manufacturing Operations

**DOI:** 10.1002/ieam.4141

**Published:** 2019-04-29

**Authors:** Joan Tell, Daniel J Caldwell, Andreas Häner, Jutta Hellstern, Birgit Hoeger, Romain Journel, Frank Mastrocco, Jim J Ryan, Jason Snape, Jürg Oliver Straub, Jessica Vestel

**Affiliations:** ^1^ Merck & Co Kenilworth New Jersey USA; ^2^ Johnson & Johnson New Brunswick New Jersey USA; ^3^ F Hoffmann‐La Roche Basel Switzerland; ^4^ Novartis Pharma AG Basel Switzerland; ^5^ Sanofi Gentilly France; ^6^ Pfizer Inc, New York New York USA; ^7^ GlaxoSmithKline Ware United Kingdom; ^8^ AstraZeneca Cheshire United Kingdom

**Keywords:** Antibiotic, Antimicrobial resistance (AMR), Pharmaceutical resistance, PNEC, Antibiotic resistance

## Abstract

In 2016, the United Nations declared the need for urgent action to combat the global threat of antimicrobial resistance (AMR). In support of this effort, the pharmaceutical industry has committed to measures aimed at improving the stewardship of antibiotics both within and outside the clinic. Notably, a group of companies collaborated to specifically address concerns related to antibiotic residues being discharged from manufacturing sites. In addition to developing a framework of minimum environmental expectations for antibiotic manufacturers, science‐based receiving water targets were established for antibiotics discharged from manufacturing operations. This paper summarizes the holistic approach taken to derive these targets and includes previously unpublished, company‐generated, environmental toxicity data.

## Key Points

- A recommended approach for assessing the risks from antibiotic manufacturing discharges is presented that considers both environmental endpoints and antimicrobial resistance.
- Also included are previously unpublished industry data on environmental toxicity of antibiotics.

Antibiotic resistance represents a severe and increasing global threat to human health, so much so that in 2016, during the 71^st^ session of the General Assembly (UNGA), the United Nations Member States adopted an antimicrobial resistance (AMR) declaration, only the fourth health issue to reach the UNGA agenda in its history. Tackling the challenge of antibiotic resistance requires a “one‐health perspective” that considers 1) human health, 2) animal health, and 3) the environmental dimension of AMR. It is crucial that all stakeholders be involved in effective stewardship across antibiotic production, use, and disposal.

As a mechanism to survive in the presence of toxic environmental factors, bacteria develop resistance naturally via spontaneous mutation and/or through acquisition of genetic determinants from other bacteria (Munita and Arias 2016). Patient use of antibiotics for viral infections or other ailments not caused by bacteria, as well as their use of incorrect dosages, exposes bacteria needlessly to antibiotics. This exposure presents the selective pressure necessary to allow antibiotic‐resistant bacteria to emerge. In addition, antibiotic use in animals as a protective measure or to promote growth increases human antibiotic exposure through the food chain and the environment from animal waste.

The elevated presence of antibiotics in the environment is believed to be increasing the rate of antibiotic resistance selection (Wright 2010; Finley et al. 2013; Bengtsson‐Palme et al. 2018), although the relationship and significance of environmental reservoirs of resistance and adverse human health impacts is still under investigation. Current evidence links the overall global distribution of antibiotics released to the environment to patient and animal excretion (Boxall et al. 2012). Manufacturing effluents from antibiotic production sites also have been postulated to contribute to AMR development locally (Kleywegt et al. 2019; Larsson 2014; Larsson et al. 2007, 2018). Thus, given the seriousness of the health threat, all sources of antibiotics to the environment are being examined and reductions being sought as part of this “one health” approach.

The use of appropriate measures based on risk to adequately control manufacturing effluent emissions is a priority for the pharmaceutical industry and is an approach already adopted by several companies (Caldwell et al. 2016). This is in line with the Wellcome Trust Review on Antimicrobial Resistance, which recommends the setting of minimum standards for manufacturing of antibiotics based on the current state of the science (O'Neill 2016).

At the UN High‐Level Meeting on Antimicrobial Resistance in 2016, thirteen pharmaceutical companies presented a roadmap that laid out 4 key commitments to reduce AMR and promote innovation in the field of antimicrobial chemotherapy. The commitments follow the principles in the Declaration by the Pharmaceutical, Biotechnology and Diagnostics Industries on Combating Antimicrobial Resistance (“Industry Declaration”) made at the 2016 World Economic Forum in Davos. This unprecedented collaboration marked a significant milestone in the fight against AMR (IFPMA 2016; AMR Industry Alliance 2017b).

In publishing this roadmap, the signatory companies establish their shared goals to overcome the threat of AMR. The companies are dedicated to working toward reducing the incidence of antimicrobial resistance; improving access to high‐quality antibiotics, vaccines, and diagnostics; investing in research and development; and collaborating with governments and other stakeholders to sustain those investments.

Specifically, the roadmap commits signatories to

- reduce the environmental impact from the production of antibiotics;
- help ensure antibiotics are used only by patients who need them;
- improve access to current and future antibiotics, vaccines, and diagnostics; and
- explore new opportunities for open collaborations between industry and the public sector.

More information on the commitments of the AMR industry roadmap signatories is available in the roadmap for progress report (IFPMA 2016). The signatories are aligned in their intent to build and share common practices. Specifically, the commitment to address manufacturing‐related concerns states that measures will be supported to reduce the environmental impact from production of antibiotics and the following 4 goals will be completed for each of the signatory companies:

1) Review manufacturing and supply chains to assess good practice in controlling releases of antibiotics into the environment.
2) Establish a common framework for managing antibiotic discharge, building on existing work such as the Pharmaceutical Supply Chain Initiative (PSCI Pharmaceutical Supply Chain Initiative 2019), and start to apply it across manufacturing and supply chains by 2018.
3) Work with stakeholders to develop a practical mechanism to transparently demonstrate that supply chains meet the standards in the framework.
4) Work with independent technical experts to establish science‐driven, risk‐based targets for discharge concentrations for antibiotics and good practice methods to reduce environmental impact of manufacturing discharges by 2020.

Measures to meet each of the above commitments are complete or actively ongoing. In particular, the roadmap signatories are in, or have already finalized, the process of auditing or reauditing and reviewing their supply chains for active substance production and for formulation of antibiotics with a specific focus on losses of active substances to the environment, mainly via production of wastewaters. The framework, as called for in commitment 2 is complete and has been published in the AMR Industry Alliance (2018a) report “Tracking Progress to Address AMR January 2018,” and incorporation of critical components of this framework into the PSCI audit protocol is in progress. A mechanism to transparently demonstrate progress, called for in commitment 3, is under development and will be shared in the next AMR Industry Alliance report for release in 2020.

The target of reducing production‐related losses to concentrations unlikely to result in adverse effects in the receiving environment needs to be assessed on the basis of current knowledge and understanding. The purpose of the present communication is to share science‐based targets and the empirical data behind them for antibiotic concentrations in manufacturing discharges (commitment 4).

The industry roadmap signatories convened an expert committee consisting of environmental toxicologists, risk assessors, microbiologists, and engineers from member companies, to review the state of the science and to develop recommended risk‐based targets for manufacturing discharges to reduce the potential for contributing to AMR. As a first step, it was agreed that the recommendation for targets should address not only AMR but also should protect ecological receptors in the receiving environment using traditional environmental endpoints. We believe that, given the current state of the science, the best approach is to derive predicted no‐effect concentrations (PNECs) that can be applied in the receiving stream. Tolerable discharge targets could then be derived in a site‐specific manner that considers characteristics of the local environment (e.g., wastewater treatment capabilities, stream flow, location of receptors).

Leveraging the existing data generated in support of regulatory drug approvals in the European Union (EMA European Medicines Agency 2006) and other company voluntary programs, environmental toxicity data were compiled from both industry studies provided by roadmap members and from the peer‐reviewed literature when studies were deemed to be reliable. Preference was given to data generated following recognized Organisation for Economic Co‐operation and Development (OECD) guidelines, or studies of a similar nature, and studies performed in compliance with the OECD Principles of Good Laboratory Practice (GLP).

Environmental predicted no‐effect concentration (PNEC‐ENV) values were derived from toxicity endpoint data with an assessment factor applied, consistent with European guidance (ECHA European Chemicals Agency 2008; EU WFD European Union Water Framework Directive 2018). Because cyanobacteria are considered most sensitive to antibiotics (EMA European Medicines Agency 2006; LePage et al. 2017), data sets were considered complete if cyanobacteria data were available following the OECD 201 guideline, or equivalent (OECD 2011). If cyanobacteria data were not available, the lowest chronic no observed effect concentration (NOEC) or 10% effect concentration (EC10) was used when chronic data for 3 trophic levels were available. In those cases, PNECs were marked to identify the lack of cyanobacteria results (Table 1 and Supplemental Data). In general, antibiotics are not particularly toxic to humans or other vertebrates. Therefore, provided that there is good evidence for lack of mammalian toxicity, an assessment factor of 10 may reasonably be applied to the lowest chronic NOEC or EC10 of cyanobacterial, green algal, and daphnid tests even in the absence of fish data (Baumann et al. 2015). For consistency, where new industry testing has been initiated, cyanobacteria is the indicator organism of choice for conducting robust environmental risk assessment on antibiotics.

**Table 1:** Environmental toxicity, PNEC‐ENV, and PNEC‐MIC values

	Lowest NOEC/EC10	PNEC‐ENV	PNEC‐MIC	Lowest PNEC
Active pharmaceutical ingredient	(µg/L)	(µg/L)	(µg/L)	(µg/L)
Amikacin	N/A	N/A	16	16
Amoxicillin	Testing ongoing	N/A	0.25	0.25
Amphotericin B	N/A	N/A	0.02	0.02
Ampicillin	8.7	0.87	0.25	0.25
Anidulafungin	N/A	N/A	0.02	0.02
Avibactam	2000^a^	200	N/A	200
Avilamycin	N/A	N/A	8.0	8.0
Azithromycin	0.2	0.02	0.25	0.02
Aztreonam	N/A	N/A	0.5	0.5
Bacitracin	N/A	N/A	8.0	8.0
Bedaquiline	0.8	0.08	N/A	0.08
Benzylpenicillin	N/A	N/A	0.25	0.25
Capreomycin	N/A	N/A	2.0	2.0
Cefaclor	N/A	N/A	0.50	0.50
Cefadroxil	Testing ongoing	N/A	2.0	2.0
Cefalonium	211	21	N/A	21
Cefaloridine	N/A	N/A	4.0	4.0
Cefalothin	N/A	N/A	2.0	2.0
Cefazolin	N/A	N/A	1.0	1.0
Cefdinir	N/A	N/A	0.25	0.25
Cefepime	N/A	N/A	0.5	0.5
Cefixime	1.8	0.18	0.06	0.06
Cefoperazone	N/A	N/A	0.5	0.5
Cefotaxime	1.0	0.1	0.13	0.1
Cefoxitin	N/A	N/A	8.0	8.0
Cefpirome	N/A	N/A	0.06	0.06
Cefpodoxime	N/A	N/A	0.25	0.25
Cefquinome	16	1.6	N/A	1.6
Ceftaroline	1.2	0.12	0.06	0.06
Ceftazidime	13	1.3	0.5	0.5
Ceftibuten	N/A	N/A	0.25	0.25
Ceftiofur	N/A	N/A	0.06	0.06
Ceftobiprole	2.3	0.23	0.25	0.23
Ceftolozane	19	1.9	N/A	1.9
Ceftriaxone	100	10	0.03	0.03
Cefuroxime	8.4	0.84	0.5	0.5
Cephalexin	0.77	0.08	4.0	0.08
Cephradine	Testing ongoing	N/A	N/A	N/A
Chloramphenicol	N/A	N/A	8.0	8.0
Ciprofloxacin	5.65	0.57	0.06	0.06
Clarithromycin	0.8	0.08	0.25	0.08
Clinafloxacin	N/A	N/A	0.5	0.5
Clindamycin	1.0	0.1	1.0	0.1
Cloxacillin	Testing ongoing	N/A	0.13	0.13
Colistin	90	9.0	2.0	2.0
Daptomycin	3900	390	1.0	1.0
Delamanid	0.3	0.03	N/A	0.03
Doripenem	1.1	0.11	0.13	0.11
Doxycycline	Testing ongoing	N/A	2.0	2.0
Enramycin	48	4.8	N/A	4.8
Enrofloxacin	19	1.9	0.06	0.06
Ertapenem	140	14	0.13	0.13
Erythromycin	5.0	0.5	1.0	0.5
Ethambutol	N/A	N/A	2.0	2.0
Faropenem	N/A	N/A	0.02	0.02
Fidaxomicin	5800	580	0.02	0.02
Florfenicol	N/A	N/A	2.0	2.0
Fluconazole	N/A	N/A	0.25	0.25
Flumequine	N/A	N/A	0.25	0.25
Fosfomycin	N/A	N/A	2.0	2.0
Fusidic acid	N/A	N/A	0.5	0.5
Gatifloxacin	N/A	N/A	0.13	0.13
Gemifloxacin	N/A	N/A	0.06	0.06
Gentamicin	1.5	0.15	1.0	0.15
Imipenem	4.1	0.41	0.13	0.13
Isoniazid	N/A	N/A	0.13	0.13
Itraconazole	N/A	N/A	0.01	0.01
Kanamycin	10.5	1.0	2.00	1.0
Levofloxacin	Testing ongoing	N/A	0.25	0.25
Lincomycin	8.1	0.81	2.0	0.81
Linezolid	67	6.7	8.0	6.7
Loracarbef	N/A	N/A	2.0	2.0
Mecillinam	N/A	N/A	1.0	1.0
Meropenem	15	1.5	0.06	0.06
Metronidazole	N/A	N/A	0.13	0.13
Minocycline	Testing ongoing	N/A	1.0	1.0
Moxifloxacin	N/A	N/A	0.13	0.13
Mupirocin	N/A	N/A	0.25	0.25
Nalidixic acid	N/A	N/A	16	16
Narasin	N/A	N/A	0.5	0.5
Neomycin	0.3	0.03	2.0	0.03
Netilmicin	N/A	N/A	0.5	0.5
Nitrofurantoin	N/A	N/A	64	64
Norfloxacin	1200	120.00	0.5	0.5
Ofloxacin	100	10.00	0.5	0.5
Oxacillin	N/A	N/A	1.0	1.0
Oxytetracycline	180	18	0.5	0.5
Pefloxacin	N/A	N/A	8.0	8.0
Phenoxymethylpenicillin	N/A	N/A	0.06	0.06
Piperacillin	N/A	N/A	0.5	0.5
Polymixin B	0.57	0.06	N/A	0.06
Retapamulin	N/A	N/A	0.06	0.06
Rifampicin	N/A	N/A	0.06	0.06
Roxithromycin	68.4	6.8	1.0	1.0
Secnidazole	N/A	N/A	1.0	1.0
Sparfloxacin	N/A	N/A	0.06	0.06
Spectinomycin	N/A	N/A	32	32
Spiramycin	10.9	1.1	0.5	0.5
Streptomycin	N/A	N/A	16	16
Sulbactam	N/A	N/A	16	16
Sulfadiazine	130	13	N/A	13
Sulfamethoxazole	5.9	0.6	16	0.6
Tedizolid	31.8	3.2	N/A	3.2
Teicoplanin	N/A	N/A	0.5	0.5
Telithromycin	N/A	N/A	0.06	0.06
Tetracycline	32	3.2	1.0	1.0
Thiamphenicol	N/A	10	1.0	1.0
Tiamulin	N/A	N/A	1.0	1.0
Ticarcillin	N/A	N/A	8.0	8.0
Tigecycline	2^a^	2.0	1.0	1.0
Tildipirosin	4.2	0.42	N/A	0.42
Tilmicosin	N/A	N/A	1.0	1.0
Tobramycin	51	5.1	1.0	1.0
Trimethoprim	1000	100	0.5	0.5
Trovafloxacin	N/A	N/A	0.03	0.03
Tylosin	10	1.0	4.0	1.0
Vancomycin	N/A	N/A	8.0	8.0
Viomycin	N/A	N/A	2.0	2.0
Virginiamycin	N/A	N/A	2.00	2.0

N/A = not applicable; NOEC = no observed effect concentration; PNEC‐ENV = environmental predicted no‐effect concentration; PNEC‐MIC = minimum inhibitory predicted no‐effect concentration.

^a^ Indicates cyanobacteria data not available.

The next step was to assess the different options for deriving PNECs that would be protective against the spread of AMR. Approaches to reduce AMR risk presented in the literature (Bengtsson‐Palme and Larsson 2016; Gullberg et al. 2011; Kümmerer and Henninger 2003; Le Page et al. 2017) as well as discussions at key scientific meetings on the topic (AMR Industry Alliance 2018b; EDAR Environmental Dimension of Antibiotic Resistance, 2017; Jones et al. 2018) were considered.

In the following list, we summarize the different approaches:

1) A key performance measure of antibiotic effectiveness is the minimum inhibitory concentration (MIC). The MIC is the lowest concentration of an antibiotic that inhibits 100% of the visible growth of a given strain of bacteria after 24‐h incubation. The MICs are measured in clinically relevant bacteria and documented in the European Committee on Antimicrobial Susceptibility Testing database (EUCAST European Committee on Antimicrobial Susceptibility Testing 2013). These data are useful in extrapolation to a PNEC for resistance, although it is recognized that resistance may occur at concentrations below the MIC (Gullberg et al. 2011).
  Bengtsson‐Palme and Larsson (2016) recommend an approach for deriving PNECs by extrapolating MIC data from the EUCAST database and applying an additional safety factor to derive PNECs for resistance. To date, this is the sole source of published PNECs for antibiotics that are specifically derived as a proxy to address resistance. We designate these as PNEC‐MIC. Comparison of these values to the available literature for microbial populations in natural conditions suggests that these published PNECs are a conservative estimate of the target for protection against resistance. However, it can be noted that NOECs or EC10 values derived for some species, especially cyanobacteria used to derive a PNEC‐ENV, may be lower than proposed criteria designed to be protective of AMR (i.e., the PNEC‐MIC) (Le Page et al. 2017) (Table 1).
2) Another performance measure of antibiotic effectiveness is the minimum selective concentration (MSC). The MSC is defined as the minimum concentration at which the presence and expression of resistance genes provide bacteria an advantage due to fitness over nonresistance strains of the same species or strain (Le Page et al. 2017). The MSC is a theoretical threshold that is determined in the laboratory for any microorganism and antibiotic pair (Singer et al. 2016). However, to date there is a paucity of MSC data to derive PNECs for the majority of antibiotics and no standardized and validated approach to determine an MSC. Additionally, it is unclear whether these single‐species standard lab tests are relevant to environmental exposures involving microbial populations in natural conditions (Murray et al. 2018). Methods to determine MSCs also require a priori knowledge of the resistance mechanisms and will not capture unknown types of AMR.
3) Microbial communities cannot confer resistance without the dissemination of antibiotic resistance genes (ARGs) or mobile genetic elements (MGEs), and it has been proposed that ARGs are a form of genetic pollution in their own right (Pruden et al. 2006). As such, measurements of ARGs and MGEs in the environment could lend insight into the potential for gene transfer and for other members of a microbial community to become resistant. However, quantifying the environmental and human health risks associated with the relative abundance of specific ARGs within a given environmental sample or discharge requires further investigation before they can be used to define environmental protection goals (Smalla et al. 2018). Further research is also required to determine the dominant routes for transfer of ARGs between environmental and pathogenic bacteria such that they may cause resistant infectious disease in humans. Jechalke et al. (2014) concluded that the amount of bacteria carrying transferable ARGs is higher when exposed to pollutants, and that when selective pressures are reduced or eliminated, the proportion of the population that carries antibiotic resistance plasmids is reduced. Additional research on ARGs and MGEs is recommended by Larsson et al. (2018), specifically to gain better understanding of the relative contributions of various sources of antibiotics and antibiotic resistant bacteria in the environment, as well as how anthropogenic sources can impact the evolution of resistance.

Considering the information presently available and the urgent need for action, it is our view that both the environmental PNECs (PNEC‐ENVs) and the PNECs developed by Bengtsson‐Palme and Larsson (2016) (PNEC‐MICs) should be considered when assessing discharges at antibiotic production facilities. We recommend using the lower of the 2 values to be protective of our ecological resources, and also to lower the pressure for the evolution, selection, and maintenance of AMR in the environment (Table 1). It is also clear that in order to meet many of these PNEC values, best practices as presented in the framework (commitment 2) will need to be implemented.

xWe also advocate that the comparison point is the predicted or measured concentration in the receiving aquatic environment, which is consistent with current practices (EU WFD European Union Water Framework Directive 2018). Using the wastewater treatment plant as the assessment point makes less sense to us because it would preclude the opportunity for treatment to partially or fully degrade those antibiotics that are known to be removed at least in part during wastewater treatment and would limit the potential for AMR selection to a “contained” or “engineered” environment.

These recommended target concentrations are being shared publicly via the AMR Alliance webpage (AMR Industry Alliance 2017a). It is important to reiterate that the values are recommended based on currently available information and, thus, may change as new information comes to light. Therefore, they should not be considered definitive and users should remain flexible and aware, considering changes in our understanding of how AMR occurs and the role of environment. Besides their use for manufacturing operations, these PNECs may also inform risk assessments in other environmental domains such as municipal or agricultural wastes with due consideration of other contributions to AMR above and beyond the presence of antibiotics alone (e.g., metals or biocides).

In conclusion, the publication of these targets fulfills a critical societal need identified by the United Nations and collaborating organizations to address some of the environmental concerns posed by AMR. We welcome opportunities to engage in scientific discussion with all stakeholders with the aims of expanding the knowledge base, developing and improving ways to assess risks, and optimizing strategies to deal with AMR, without compromising patient access to necessary medicines.

## Data Accessibility

All data are available by contacting the corresponding author, Joan Tell, at joan_tell@merck.com.

## SUPPLEMENTAL DATA

Detailed environmental toxicity data from unpublished studies and relevant literature to support the work.

## Supporting information

This article contains online‐only Supplemental Data.

Supporting information
