# Supplementary material for: Science‐based Targets for Antibiotics in Receiving Waters from Pharmaceutical Manufacturing Operations
Source: Integr Environ Assess Manag. 2019 Apr 29;15(3):312–9. doi: 10.1002/ieam.4141 (PMC6849714; doi:10.1002/ieam.4141)
Supplement: Supplementary file 1 — Supporting information [file IEAM-15-312-s001.docx]

# Supplementary Data Table

| **Active Pharmaceutical Ingredient** | **NOEC / EC_10_ (µg/L)** | **Species** | | **Test Guideline / Reference** | **PNEC_ENV_ (µg/L)** | **PNEC_ENV_ Rationale** |
| --- | --- | --- | --- | --- | --- | --- |
| Amoxicillin | N/A | *Anabaena flos-aquae* | | OECD 201 | N/A | Industry testing on-going |
|  | *530000* | | *Raphidocelis subcapitata* | OECD 201 |  |  |
| Ampicillin | 8.7 | *Synechococcus leopoliensis* | | OECD 201 | 0.87 | *Synechococcus leopoliensis* NOEC÷10 |
|  | 13 | *Anabaena flos-aquae* | | OECD 201 |  |  |
|  | 94300 | *Desmodesmus subspicatus* | | OECD 201 |  |  |
|  | 100000 | *Raphidocelis subcapitata* | | OECD 201 |  |  |
|  | 91500 | *Daphnia magna* | | OECD 211 |  |  |
| Avibactam | 120000 | *Raphidocelis subcapitata* | | OECD 201 | 200* | *Pimephales promelas* NOEC÷10 |
|  | 2000 | *Pimephales promelas* | | OECD 210 |  |  |
|  | 100000 | *Daphnia magna* | | OECD 211 |  |  |
| Azithromycin | 0.19 | *Microcystis aeruginosa* | | OECD 201 | 0.02 | *Microcystis aeruginosa* NOEC÷10 |
|  | 1.8 | *Raphidocelis subcapitata* | | OECD 201 |  |  |
|  | 4.4 | *Ceriodaphnia dubia* | | EPA 1002.0 |  |  |
|  | 4600 | *Pimephales promelas* | | OECD 210 |  |  |
| Bedaquiline | 0.8 | *Anabaena flos-aquae* | | OECD 201 | 0.08 | *Anabaena flos-aquae* NOEC÷10 |
|  | 0.8 | *Raphidocelis subcapitata* | | OECD 201 |  |  |
|  | 4.1 | *Brachydanio rerio* | | OECD 210 |  |  |
|  | 4.7 | *Daphnia magna* | | OECD 211 |  |  |
| Cefadroxil | N/A | *Anabaena flos-aquae* | | OECD 201 | N/A | Industry testing on-going |
| Cefalonium | 211 | *Anabaena flos-aquae* | | OECD 201 | 21.10 | *Anabaena flos-aquae* NOEC÷10 |
|  | 91000 | *Raphidocelis subcapitata* | | OECD 201 |  |  |
| Cefixime | 1.8 | *Anabaena flos-aquae* | | OECD 201 | 0.18 | *Anabaena flos-aquae* NOEC÷10 |
| Cefotaxime | 0.98 | *Anabaena flos-aquae* | | OECD 201 | 0.10 | *Anabaena flos-aquae* NOEC÷10 |
| Cefquinome | 16 | *Anabaena flos-aquae* | | OECD 201 | 1.60 | *Anabaena flos-aquae* NOEC÷10 |
| Ceftaroline | 1.2 | *Anabaena flos-aquae* | | OECD 201 | 0.12 | *Anabaena flos-aquae* NOEC÷10 |
|  | 33000 | *Raphidocelis subcapitata* | | OECD 201 |  |  |
|  | 5000 | *Pimephales promelas* | | OECD 210 |  |  |
|  | 7900 | *Daphnia magna* | | OECD 211 |  |  |
| Ceftazidime | 13 | *Anabaena flos-aquae* | | OECD 201 | 1.30 | *Anabaena flos-aquae* NOEC÷10 |
|  | 120000 | *Raphidocelis subcapitata* | | OECD 201 |  |  |
|  | 8000 | *Pimephales promelas* | | OECD 210 |  |  |
|  | 9200 | *Daphnia magna* | | OECD 211 |  |  |
| Ceftobiprole | 2.3 | *Anabaena flos-aquae* | | OECD 201 | 0.23 | *Anabaena flos-aquae* NOEC÷10 |
|  | 5600 | *Brachydanio rerio* | | OECD 210 |  |  |
|  | 37000 | *Daphnia magna* | | OECD 211 |  |  |
| Ceftolozane | 19 | *Anabaena flos-aquae* | | OECD 201 | 1.90 | *Anabaena flos-aquae* NOEC÷10 |
|  | 10000 | *Pimephales promelas* | | OECD 210 |  |  |
|  | 9600 | *Daphnia magna* | | OECD 211 |  |  |
| Ceftriaxone | 100 | *Synechococcus leopoliensis* | | OECD 201 | 10.00 | *Synechococcus leopoliensis* NOEC÷10 |
|  | 98800 | *Raphidocelis subcapitata* | | OECD 201 |  |  |
|  | 28500 | *Daphnia magna* | | OECD 211 |  |  |
|  |  |  | |  |  |  |
| Cefuroxime | 8.4 | *Anabaena flos-aquae* | | OECD 201 | 0.84 | *Anabaena flos-aquae* NOEC÷10 |
|  | 91000 | *Raphidocelis subcapitata* | | OECD 201 |  |  |
| Cephalexin | 0.77 | *Anabaena flos-aquae* | | OECD 201 | 0.08 | *Anabaena flos-aquae* NOEC÷10 |
|  | 120000 | *Raphidocelis subcapitata* | | OECD 201 |  |  |
| Cephradine | N/A | *Anabaena flos-aquae* | | OECD 201 | N/A | Industry testing on-going |
| Ciprofloxacin | 5.65 | *Anabaena flos-aquae* | | (Ebert et al. 2011) | 0.57 | *Anabaena flos-aquae* EC_10_÷10 |
|  | ≥8042 | *Desmodesmus subspicatus* | | (Ebert et al. 2011) |  |  |
| Clarithromycin | <0.8 | *Anabaena flos-aquae* | | (Baumann et al. 2015) | 0.08 | *Anabaena flos-aquae* NOEC÷10 |
| Clindamycin | 8.1 | *Anabaena flos-aquae* | | OECD 201 | 0.10 | *Raphidocelis subcapitata* NOEC÷10 |
|  | 0.98 | *Raphidocelis subcapitata* | | OECD 201 |  |  |
| Cloxacillin | N/A | *Anabaena flos-aquae* | | OECD 201 | N/A | Industry testing on-going |
| Colistin | 90 | *Anabaena flos-aquae* | | OECD 201 | 9.00 | *Anabaena flos-aquae* EC_10_÷10 |
| Daptomycin | 17000 | *Anabaena flos-aquae* | | OECD 201 | 390.00 | *Pimephales promelas* NOEC÷10 |
|  | 34000 | *Raphidocelis subcapitata* | | OECD 201 |  |  |
|  | 3900 | *Pimephales promelas* | | OECD 210 |  |  |
|  | 6800 | *Daphnia magna* | | OECD 211 |  |  |
| Delamanid | ≥0.42 | *Anabaena flos-aquae* | | OECD 201 | 0.03 | *Raphidocelis subcapitata* NOEC÷10 |
|  | ≥0.3 | *Raphidocelis subcapitata* | | OECD 201 |  |  |
|  | ≥0.71 | *Pimephales promelas* | | OECD 210 |  |  |
|  | ≥0.59 | *Daphnia magna* | | OECD 211 |  |  |
| Doripenem | 1.1 | *Anabaena flos-aquae* | | OECD 201 | 0.11 | *Anabaena flos-aquae* NOEC÷10 |
|  | 61000 | *Scenedesmus subspicatus* | | OECD 201 |  |  |
|  | 9200 | *Brachydanio rerio* | | OECD 210 |  |  |
|  | 9400 | *Daphnia magna* | | OECD 211 |  |  |
| Doxycycline | N/A | *Anabaena flos-aquae* | | OECD 201 | N/A | Industry testing on-going |
| Enramycin | 48 | *Anabaena flos-aquae* | | OECD 201 | 4.80 | *Anabaena flos-aquae* NOEC÷10 |
|  | 960 | *Raphidocelis subcapitata* | | OECD 201 |  |  |
| Enrofloxacin | 19.1 | *Anabaena flos-aquae* | | (Ebert et al. 2011) | 1.91 | *Anabaena flos-aquae* EC_10_ ÷10 |
|  | 500 | *Desmodesmus subspicatus* | | (Ebert et al. 2011) |  |  |
| Ertapenem | 140 | *Anabaena flos-aquae* | | OECD 201 | 14.00 | *Anabaena flos-aquae* EC_10_÷10 |
|  | 51000 | *Raphidocelis subcapitata* | | OECD 201 |  |  |
|  | 2500 | *Pimephales promelas* | | OECD 210 |  |  |
|  | 82000 | *Daphnia magna* | | OECD 211 |  |  |
| Erythromycin | 5 | *Anabaena flos-aquae* | | (Gonzalez-Pleiter et al. 2013) | 0.50 | *Anabaena flos-aquae* EC_10_÷10 |
|  | 36 | *Raphidocelis subcapitata* | | (Gonzalez-Pleiter et al. 2013) |  |  |
| Fidoxamicin | 5800 | *Anabaena flos-aquae* | | OECD 201 | 580.00 | *Anabaena flos-aquae* NOEC÷10 |
|  | 8910 | *Pimephales promelas* | | OECD 210 |  |  |
|  | 19600 | *Daphnia magna* | | OECD 211 |  |  |
| Gentamicin | 2 | *Anabaena flos-aquae* | | OECD 201 | 0.15 | *Raphidocelis subcapitata* EC_10_÷10 |
|  | 1.5 | *Raphidocelis subcapitata* | | OECD 201 |  |  |
|  | 6.2 | *Daphnia magna* | | OECD 211 |  |  |
| Imipenem | 4.1 | *Anabaena flos-aquae* | | OECD 201 | 0.41 | *Anabaena flos-aquae* EC_10_÷10 |
|  | 74000 | *Raphidocelis subcapitata* | | OECD 201 |  |  |
| Kanamycin | 11 | *Anabaena flos-aquae* | | OECD 201 | 1.05 | *Synechococcus leopoliensis* EC_10_÷10 |
|  | 310 | *Raphidocelis subcapitata* | | OECD 201 |  |  |
|  | 10.5 | *Synechococcus leopoliensis* | | OECD 201 |  |  |
| Levofloxacin | N/A | *Anabaena flos-aquae* | | OECD 201 | N/A | Industry testing on-going |
| Lincomycin | 12.20 | *Anabaena flos-aquae* | | (Guo et al. 2016) | 0.81 | *Synechococcus leopoliensis* EC_10_÷10 |
|  | 77.24 | *Desmodesmus subspicatus* | | (Guo et al. 2016) |  |  |
|  | 357.75 | *Raphidocelis subcapitata* | | (Guo et al. 2016) |  |  |
|  | 8.13 | *Synechococcus leopoliensis* | | (Guo et al. 2016) |  |  |
| Linezolid | 67 | *Anabaena flos-aquae* | | OECD 201 | 6.70 | *Anabaena flos-aquae* NOEC÷10 |
|  | 9900 | *Pimephales promelas* | | OECD 210 |  |  |
|  | 24000 | *Daphnia magna* | | OECD 211 |  |  |
| Meropenem | 15 | *Anabaena flos-aquae* | | OECD 201 | 1.50 | *Anabaena flos-aquae* NOEC÷10 |
| Minocycline | N/A | *Anabaena flos-aquae* | | OECD 201 | N/A | Industry testing on-going |
|  | 2030 | *Chlorella spp.* | | Internal study |  |  |
| Neomycin | 0.3 | *Anabaena flos-aquae* | | OECD 201 | 0.03 | *Anabaena flos-aquae* EC_10_÷10 |
|  | 2.2 | *Raphidocelis subcapitata* | | OECD 201 |  |  |
| Norfloxacin | 1200 | *Anabaena flos-aquae* | | (Gonzalez-Pleiter et al. 2013) | 120.00 | *Anabaena flos-aquae* EC_10_÷10 |
|  | 10900 | *Raphidocelis subcapitata* | | (Gonzalez-Pleiter et al. 2013) |  |  |
| Ofloxacin | 100 | *Anabaena flos-aquae* | | OECD 201 | 10.00 | *Anabaena flos-aquae* NOEC÷10 |
| Oxytetracycline | 180 | *Raphidocelis subcapitata* | | (Ando et al. 2007) | 18.00 | *Raphidocelis subcapitata* NOEC÷10 |
|  | 1500 | *Anabaena flos-aquae* | | (Kolar et al. 2014) |  |  |
| Polymixin B | 0.57 | *Anabaena flos-aquae* | | OECD 201 | 0.06 | *Anabaena flos-aquae* EC_10_÷10 |
|  | 7.6 | *Raphidocelis subcapitata* | | OECD 201 |  |  |
| Roxithromycin | 68.4 | *Anabaena flos-aquae* | | OECD 201 | 6.80 | *Anabaena flos-aquae* EC_10_ ÷ 10 |
| Spiramycin | 10.9 | *Anabaena flos-aquae* | | OECD 201 | 1.00 | *Anabaena flos-aquae* EC_10_ ÷ 10 |
| Sulfadiazine | 7200 | *Anabaena flos-aquae* | | OECD 201 | 13.00 | *Raphidocelis subcapitata* NOEC÷10 |
|  | 130 | *Raphidocelis subcapitata* | | OECD 201 |  |  |
| Sulfamethoxazole | 1250 | *Raphidocelis subcapitata* | | (Ferrari et al. 2004) | 0.60 | *Synechococcus leopoliensis* NOEC÷10 |
|  | 5.9 | *Synechococcus leopoliensis* | | (Ferrari et al. 2004) |  |  |
| Tedizolid | 98 | *Anabaena flos-aquae* | | OECD 201 | 3.20 | *Anabaena flos-aquae* EC_10_÷10 |
|  | 31.75 | *Pimephales promelas* | | OECD 210 |  |  |
|  | 600 | *Daphnia magna* | | OECD 211 |  |  |
| Tetracycline | 2500 | *Anabaena flos-aquae* | | (Gonzalez-Pleiter et al. 2013) | 3.20 | *Raphidocelis subcapitata* EC_10_÷10 |
|  | 32 | *Raphidocelis subcapitata* | | (Gonzalez-Pleiter et al. 2013) |  |  |
| Tigecycline | 1650† | *Raphidocelis subcapitata* | | OECD 201 | 2.20* | *Pimephales promelas* NOEC÷10 |
|  | 22 | *Pimephales promelas* | | OECD 210 |  |  |
|  | 2100 | *Daphnia magna* | | OECD 211 |  |  |
| Tildipirosin | 4.2 | *Anabaena flos-aquae* | | OECD 201 | 0.42 | *Anabaena flos-aquae* EC_10_÷10 |
|  | 47 | *Raphidocelis subcapitata* | | OECD 201 |  |  |
| Tobramycin | 51 | *Anabaena flos-aquae* | | OECD 201 | 5.10 | *Anabaena flos-aquae* NOEC÷10 |
|  | 10000 | *Pimephales promelas* | | OECD 210 |  |  |
|  | 360 | *Daphnia magna* | | OECD 211 |  |  |
| Trimethoprim | 1000 | *Anabaena flos-aquae* | | OECD 201 | 100.00 | *Anabaena flos-aquae* NOEC÷10 |
|  | 32000 | *Raphidocelis subcapitata* | | OECD 201 |  |  |
|  | 100000 | *Brachydanio rerio* | | OECD 210 |  |  |
|  | 5600 | *Daphnia magna* | | EPA 1002.0 |  |  |
| Tylosin | 18.32 | *Anabaena flos-aquae* | | (Guo et al. 2016) | 1.00 | *Synechococcus leopoliensis* EC_10_÷10 |
|  | 3710.21 | *Desmodesmus subspicatus* | | (Guo et al. 2016) |  |  |
|  | 833.65 | *Raphidocelis subcapitata* | | (Guo et al. 2016) |  |  |
|  | 10.08 | *Synechococcus leopoliensis* | | (Guo et al. 2016) |  |  |

* Indicates cyanobacteria data not available

† Indicates EC_50_ value

*Note.* In general, antibiotics are not toxic to humans or other vertebrates. Therefore, as long as there is good evidence for mammalian nontoxicity, an assessment factor of 10 may reasonably be applied to the lowest chronic NOEC of cyanobacterian, green algal and daphnid tests even in the absence of fish data (Baumann et al. 2015). Aquatic toxicity of the macrolide antibiotic clarithromycin and its metabolites. Chemosphere 120:192–198].
